# Intracranial mechanical thrombectomy of large vessel occlusions in the posterior circulation using SAVE

**DOI:** 10.1186/s12883-019-1428-x

**Published:** 2019-08-16

**Authors:** Volker Maus, Hanna Styczen, Jan Liman, Ilko Maier, Alex Brehm, Ioannis Tsogkas, Marios-Nikos Psychogios

**Affiliations:** 1 Department of Diagnostic and Interventional Neuroradiology, University Medical Center Goettingen, Goettingen, Germany; 2 Institute of Diagnostic and Interventional Radiology, Neuroradiology and Nuclear Medicine, Knappschaftskrankenhaus Bochum, Ruhr University Bochum, In der Schornau 23-25, 44892 Bochum, Germany; 3 Department of Neurology, University Medical Center Goettingen, Goettingen, Germany; 4 Department of Neuroradiology, Clinic of Radiology & Nuclear Medicine, University Hospital Basel, Basel, Switzerland

**Keywords:** Acute ischemic stroke, Posterior circulation stroke, Mechanical thrombectomy, SAVE

## Abstract

**Background:**

Mechanical thrombectomy (MT) using stent retriever assisted vacuum-locked extraction (SAVE) is a promising method for anterior circulation strokes. We present our experience with SAVE for large vessel occlusions (LVO) of the posterior circulation.

**Methods:**

We retrospectively analyzed 66 consecutive MT patients suffering from LVO of the posterior circulation. Primary endpoints were first-pass and overall complete/near complete reperfusion, defined as a modified thrombolysis in cerebral infarction (mTICI) score of 2c and 3. Secondary endpoints contained number of passes, time interval from groin puncture to reperfusion and rate of postinterventional symptomatic intracranial hemorrhage (sICH).

**Results:**

Median age was 75 years (interquartile range (IQR) 54–81 years). Baseline median National Institutes of Health stroke scale (NIHSS) was 13 (IQR 8–21). Fifty-five (83%) patients had LVO of the basilar artery and 11 (17%) of the posterior cerebral artery. Eighteen (27%) patients were treated with SAVE and 21 (32%) with aspiration only. First pass mTICI2c or 3 and overall mTICI2c or 3 were documented in 11/18 (61%) and 14/18 (78%) with SAVE and in 4/21 (19%) and 13/21 (33%) with aspiration only. Median attempt was 1 (IQR 1–2) with SAVE and 2 (IQR 1–4) with aspiration (*p* = 0.0249). Median groin to reperfusion time did not differ significantly between groups. The rate of sICH was 5% without any complications in the SAVE cohort.

**Conclusion:**

Mechanical thrombectomy of posterior large vessel occlusions with SAVE is feasible, safe, and effective with high rates of near-complete and complete reperfusion.

## Background

Mechanical thrombectomy (MT) is the standard treatment for patients suffering from acute ischemic stroke (AIS) due to intracranial large vessel occlusion (LVO) in the anterior circulation since large randomized controlled trials (RCT) showed efficacy of this strategy over standard medical care [1]. The time window for MT has furthermore expanded from 6 up to 16–24 h in carefully selected patients as recently shown in the DAWN and DEFUSE-3 trial [2, 3]. Large vessel occlusions in the posterior circulation were excluded in the aforementioned trials, but few retrospective studies demonstrated effectiveness of MT in this cohort [4–6]. Contrary to these findings, the ENDOSTROKE registry demonstrated that recanalization did not significantly predict clinical outcome in patients with basilar artery occlusion (BAO) [7]. Nevertheless, recent guidelines included MT of LVO in the posterior circulation as reasonable option when performed at centers with appropriate expertise (Class of recommendation IIb) [8].

In recent years, thrombectomy techniques have evolved rapidly by advances in catheter technology in order to improve the rate of successful reperfusion and minimize occurrence of distal embolization. The main methods of MT for LVO include stent retriever thrombectomy with or without the use of an aspiration catheter [9] or the use of a large-bore distal access catheter alone (known as “A Direct Aspiration first Pass Technique”, ADAPT) [10]. Among the combined approaches, new techniques were developed, which differ in stent retriever placement in relation to the clot, time of aspiration and/or proximal use of balloon guide catheters with promising reperfusion results in the anterior circulation [11–13]. In this study, we present our experience with the recently introduced stent retriever assisted vacuum-locked extraction (SAVE) technique in the posterior circulation.

## Methods

We report a single-center, retrospective analysis of patients, who were treated with MT for AIS caused by LVO in the posterior circulation from March 2014 to November 2018 in our department. Baseline parameters, angiographic features, technical and clinical outcome were derived from a prospectively maintained neurointerventional database. Primary endpoints were first pass and overall complete or near-complete reperfusion, defined as the modified Thrombolysis in Cerebral Infarction (mTICI) scale score of 2c and 3 [14]. Secondary endpoints contained number of passes, time interval from groin puncture to reperfusion and rate of postinterventional symptomatic intracranial hemorrhage (sICH). According to the guidelines of the local ethic committee, approval was given for the prospective acquisition of patient data (No: 13/7/15An), which was conducted in accordance with the Declaration of Helsinki.

Following inclusion criteria were applied: clinical diagnosis of AIS due to LVO in the posterior circulation and initiation of endovascular stroke treatment with complete angiographic documentation. There were no general limitations on baseline variables such as age and National Institutes of Health Stroke Scale (NIHSS) on admission or procedural characteristics, including the use of different thrombectomy techniques and intra-arterial thrombolysis, which were left to the attending neuroradiologist’s discretion. According to neurological guidelines, patients received IVT whenever possible. Procedural angiograms were reviewed independently by experienced interventional neuroradiologists to evaluate the mTICI score before and after MT and blinded to the used MT technique. In accordance to the anterior circulation, reperfusion was understood as contrast material passes beyond the area of initial occlusion. Therefore, the territory distal to the occlusion was set as 100% and percent reperfusion was measured and translated into the mTICI scoring system. Ischemic stroke was confirmed with native computed tomography (CT) or flat panel detector CT in cases of one stop management and CTA (or multiphase FDCTA) [15]. Native CT was performed within 24 h or in case of secondary worsening. Symptomatic ICH was defined as any CT-documented hemorrhage that was temporally related to deterioration in the patient’s clinical condition and increment of ≥4 points in the NIHSS. All NIHSS and modified Rankin Score (mRS) grades were assessed by a consultant neurologist.

### Thrombectomy technique with SAVE

**Fig. 1:**
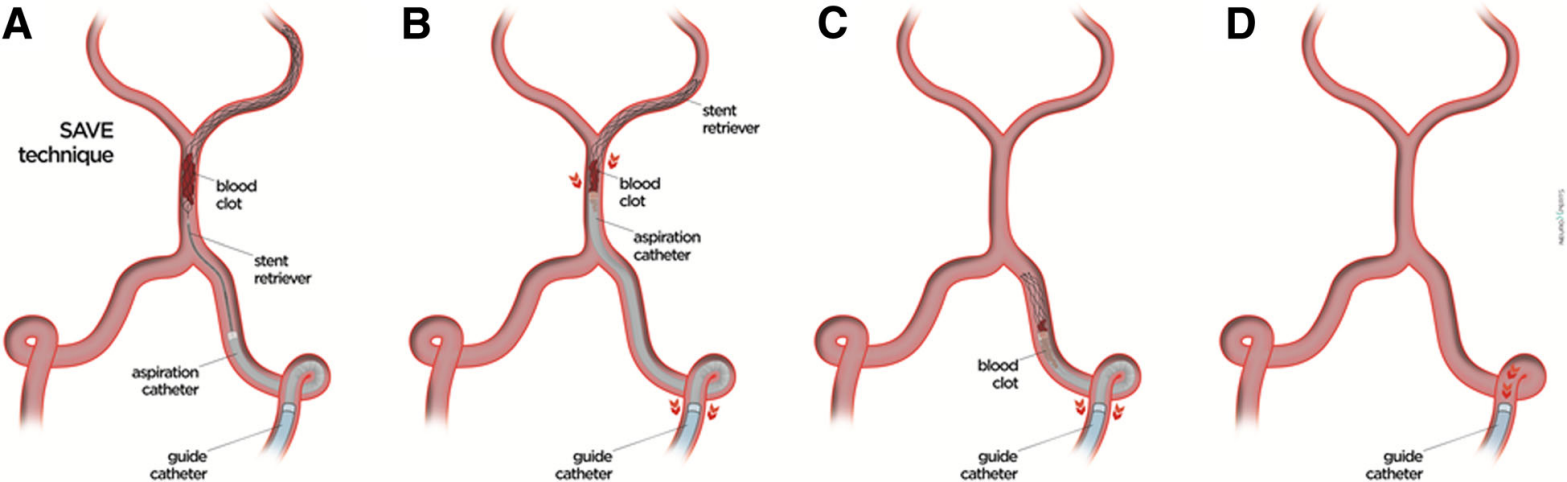
**a** The stent retriever is deployed primary distally to and with the proximal third across the occlusion site. **b** The aspiration pump is connected and activated and the tip of the aspiration catheter is advanced towards the occlusion site, while gently retracting the stent retriever until a wedge position is reached. **c** + **d** The stent retriever and aspiration catheter are removed into the guide catheter as a single unit after permanent aspiration with the pump is switched to the guide catheter while negative pressure is retained within the aspiration catheter with the use of a vacuum pressure syringe

The SAVE technique has been recently described [11]. Briefly, based on a triaxial approach, access to cerebral vasculature is obtained either with a 6 or 7 French (F) guide catheter (Envoy, Cordis, Fremont, CA, USA; Mach 1, Boston Scientific, Marlborough, MA, USA). A 0.021″ inch microcatheter is advanced through a 5F aspiration catheter (AXS Catalyst, Stryker, Fremont, CA, USA; Sofia, Microvention, Tustin, CA, USA) past the occlusion site in the P1 or P2 segment. The stent retriever (usually Trevo ProVue, Stryker) is deployed primary distally to and with the proximal third across the occlusion site. During a period of 2-8 min, while the stent retriever has the chance to integrate with the thrombus, the microcatheter is slowly retracted. The aspiration catheter is then placed to the proximal face of the thrombus and the aspiration pump is connected and activated. The tip of the aspiration catheter is advanced towards the occlusion site, while gently retracting the stent retriever until reaching a wedge position. The permanent aspiration with the pump is switched to the guide catheter while negative pressure is retained within the aspiration catheter with the use of a 60 ml vacuum pressure syringe (VacLok, Merit Medical, South Jordon, UT, USA). Then, the stent retriever and aspiration catheter are removed into the guide catheter as a single unit (Fig. 1). Pump aspiration on the guide catheter is kept 30 s after removal of the stent retriever/aspiration catheter unit in order to minimize distal emboli. Control angiograms after each pass are acquired to assess the degree of reperfusion.

### Statistical analysis

All analyses were conducted using MedCalc Statistical Software version 18 (MedCalc Software, Ostend, Belgium; http://www.medcalc.org; 2019). Descriptive statistics of normally distributed data are stated as mean and standard deviation; not normally distributed data are summarized as median and interquartile range (IQR). Differences between groups were examined using Fisher’s exact test or Mann-Whitney test. Statistical significance was defined as *p* ≤ 0.05.

## Results

Sixty-six patients were treated with MT due to LVO in the posterior circulation. Thirty-eight patients (58%) were male and median age was 75 years (IQR 54–81 years). Median baseline NIHSS was 13 (IQR 8–21) and the proportion of patients receiving IVT was 56% (Table 1). Large vessel occlusion was confirmed in the basilar artery in 55/66 (83%) and in the posterior cerebral artery (PCA, P1 segment) in 11/66 (17%) cases. Out of 66 patients, 18 (27%) patients were treated with SAVE and 21 (32%) patients were treated with aspiration only. Four out of eighteen (22%) patients treated with SAVE and 5/21 (24%) with aspiration only had occlusions in the PCA, respectively. Those groups did not differ in baseline parameters. In 11/66 (17%) patients, the stent retriever was retrieved within the aspiration catheter, which was left in place (“Solumbra” technique) and 7/66 (11%) patients were treated only with stent retriever (without aspiration catheter). Out of the remaining 9 patients, the clot could not be reached in 6 (9%) due to vessel tortuosity; of those, 2 (3%) patients received intraarterial thrombolysis. In 3 (5%) patients, recanalization was achieved due to prior IVT. One patient with primary aspiration received stent retriever rescue maneuver and 3 patients with primary SAVE received bailout intracranial stenting. There was no intra-arterial thrombolysis additional to SAVE or aspiration.

**Table 1:** Baseline characteristics

	Value
Number of patients	66
Male	38 (58%)
Age (years), median ± SD	75 ± 13
Arterial hypertension	49 (74%)
Atrial fibrillation	27 (41%)
Diabetes	18 (27%)
Dyslipidemia	33 (50%)
Smoker	9 (14%)
Baseline NIHSS score (median (IQR))	13 (8–21)
IVT	37 (56%)

*SD* = standard deviation; *NIHSS* = National Institute of Health Stroke Scale; *IQR* = interquartile range; *IVT* = intravenous thrombolysis

### Angiographic results with SAVE versus aspiration only

The primary endpoint of first pass complete or near complete reperfusion (mTICI ≥2c) was achieved in 11/18 (61%) with SAVE vs. 4/21 (19%) with aspiration (*p* = 0.0079, Table 2); complete reperfusion (mTICI 3) after a single pass was reached in 10/18 (56%) with SAVE vs. 2/21 (10%) with aspiration only (*p* = 0.0022). The rate of first pass successful reperfusion (mTICI ≥2b) was also higher in the SAVE group with 12/18 (67%) vs. aspiration group with 7/21 (33%, *p* = 0.0404). An overall rate of mTICI 2c and 3 on final angiogram was achieved in 14/18 (78%) SAVE cases and in 7/21 (33%) aspiration cases (*p* = 0.0061). A total of 12/18 (67%) SAVE patients and 3/21 (14%) aspiration patients were completely reperfused (mTICI 3) at the end of the procedure (*p* = 0.0009). The overall rate of successful reperfusion (mTICI ≥2b) was 16/18 (89%) with SAVE vs. 13/21 (62%) with aspiration. Median attempt was 1 (IQR 1–2) with SAVE and 2 (IQR 1–4) with aspiration (*p* = 0.0249). Median groin to reperfusion time did not differ significantly between SAVE and aspiration only groups (46 min (IQR 29–56) vs. 39 min (IQR 22–102); *p* = 0.9723).

**Table 2:** Angiographic results of SAVE and aspiration only

	SAVE	Aspiration only	*p*-value
Number of patients	18	21	–
First-pass reperfusion			
mTICI 3	10 (56%)	2 (10%)	**0.0022**
mTICI ≥2c	11 (61%)	4 (19%)	**0.0079**
mTICI ≥2b	12 (67%)	7 (33%)	**0.0404**
Final reperfusion			
mTICI 3	12 (67%)	3 (14%)	**0.0009**
mTICI ≥2c	14 (78%)	13 (33%)	**0.0061**
mTICI ≥2b	16 (89%)	13 (62%)	0.0576
Number of passes, median (IQR)	1 (1–2)	2 (1–4)	**0.0249**
Groin puncture – reperfusion	46 (29–56)	39 (22–102)	0.9723
(Minutes), median (IQR)			

*SAVE* = Stent retriever Assisted Vacuum-locked Extraction; *mTICI* = modified Thrombolysis in Cerebral Ischemia; *IQR* = interquartile range

All values in boldface are significant results (<0.05)

No complications occurred with SAVE. Two patients (5%) receiving aspiration only suffered from sICH. There were no statistical differences observed in median NIHSS and mRS at discharge between the two groups: median NIHSS score at discharge was 5 for both groups (SAVE, IQR 2–10; aspiration only, IQR 1–16) and favorable neurological outcome (mRS ≤ 2) was confirmed in 6/18 (33%) patients treated with SAVE and 8/21 (38%) patients treated with aspiration only. Overall, favorable outcome at discharge was 24/66 (36%) and median NIHSS score at discharge was 5 (IQR 2–12).

## Discussion

So far, there is one randomized trial, which compared the safety and efficacy of MT plus standard medical therapy vs. standard medical therapy in patients suffering from posterior circulation strokes [16]. The BEST trial was terminated early due to a high cross-over rate. But despite the overall negative results, patients treated with MT achieved better outcomes. However, this stroke cohort is associated with the poorest outcome of all stroke subtypes, resulting in a morbidity and mortality rate up to 70% [17]. The multi-center ENDOSTROKE registry showed at least that the use of stent retriever is an independent predictor of recanalization in the posterior circulation, however, recanalization itself did not predict clinical outcome in this study [7]. In our study, we analyzed the performance of the SAVE technique based on our experience with this method for the anterior circulation [11]. Similar to our previous results, we observed SAVE to be an effective and safe thrombectomy method in patients suffering from LVO in the posterior circulation.

As the central requirement of MT is a fast recanalization of the occlusion with complete reperfusion of the affected territory, new devices and different strategies were developed during the last years. Promising techniques using large-bore aspiration catheters and/or new generation stent retrievers had shown promising results with high reperfusion rates, however, those techniques were mainly applied in the anterior circulation [18, 19]. For the subgroup of posterior circulation strokes comparative analysis of different stroke techniques remain scarce. In our study, the final successful reperfusion rate (mTICI ≥2b) of 89% with SAVE is comparable to those of previous stent retriever series of 81 and 83% published in two meta-analysis of Gory et al. and Ye et al., respectively, [20, 21] and the ENDOSTROKE registry, which reported a TICI 2b/3 rate of 79% [7]. Previous studies, which were not included in the meta-analyses showed successful reperfusion in 70 and 73% of cases, respectively [22, 23]. A recent study in fact demonstrated aspiration only as the better first-line strategy for BAO patients compared to stent retriever for complete reperfusion with rates of 54 and 32%, respectively [6], and were comparable to 67% mTICI 3 results with SAVE in our study. However, another study did not show differences between aspiration only and stent retrieving with regard to complete reperfusion [21]. Unfortunately, a comparison of the first-pass results is not possible due to lack of such information in the aforementioned studies; however, first-pass reperfusion results with SAVE were promising with 56–67% (mTICI ≥2b/≥2c/3) and significantly higher compared to aspiration only, which led to a significant lower number of thrombectomy attempts with SAVE. However, it is to mention that the use of the mTICI score in the posterior circulation is not fully validated as demonstrated recently, which makes it more difficult to compare reperfusion results to those of anterior circulation strokes [24].

New aspiration catheters and aspiration pumps have been implemented with different results in achieving high and constant flows [25, 26]. In our study, there might have been bias due to the use of aspiration pumps and vacuum pressure syringes (which were used at the beginning). As shown by Nikoubashman et al. the Penumbra aspiration pump might achieve significantly lower flow compared to 60-milliliter VacLok vacuum pressure syringes, mostly due to the high resistance of the connecting tubing with diminished blood flow reversal (in the anterior circulation without balloon protection) [25].

Although the procedure times by using SAVE tended to be longer compared to aspiration only, no differences in clinical outcome at discharge were observed, which might possibly be explained by the better reperfusion results. However, as stated in the literature up to now [6], this study also did not show a technique’s clinical benefit towards a specific technique.

Effectiveness of proximal flow arrest/aspiration in the posterior circulation is matter of debate and limited to few cases [27, 28]. While in the anterior circulation the use of a balloon guide catheter with subsequent flow arrest is a predictor of first-pass success [29], in most of the patients with posterior stroke an antegrade flow in the basilar artery cannot be prevented due to maintained blood flow from the contralateral vertebral artery. Interestingly, in our cohort the rate of complete reperfusion was high, suggesting that loss of fragments during the retrieval maneuver is a rare phenomenon even when proximal aspiration is only applied in one of the feeding vertebral arteries. This emphasizes the meaning of distal protection by wedging the clot between stent retriever and aspiration catheter tip during the withdrawal as one major key element of SAVE. However, other factors such as contralateral low flow conditions due to vertebral artery aplasia or hypoplasia might also influence reperfusion success and were not considered in the analysis [30].

A limitation of our study is the retrospective design with the attendant selection bias. The small sample size limits the validity of the data. The observed differences between the techniques should be interpreted with caution as in our institute experience with SAVE is high compared to the aspiration only technique. The small sample size and missing evaluation of the aforementioned anatomic factors might prevent the conclusion that SAVE is more effective than aspiration. Clinical outcome after 90 days is missing; however, our intention was to focus on the angiographic results as complete reperfusion is a basic requirement for recovery of stroke patients. A prospective trial is warranted to demonstrate efficacy of SAVE for posterior circulation strokes.

## Conclusions

Mechanical thrombectomy of posterior large vessel occlusions with SAVE is feasible, safe, and effective with high rates of near-complete and complete reperfusion.

## Data Availability

All data generated or analysed during this study are included in this published article.

## Abbreviations

ADAPT: A direct aspiration first pass technique
BAO: Basilar artery occlusion
CT: Computed tomography
FDCT: Flat panel detector CT
IQR: Interquartile range
LVO: Large vessel occlusions
mRS: Modified Rankin Score
MT: Mechanical thrombectomy
mTICI: Modified thrombolysis in cerebral infarction
NIHSS: National Institutes of Health stroke scale
PCA: Posterior cerebral artery
RCT: Randomized controlled trials
SAVE: Stent retriever assisted vacuum-locked extraction
sICH: Symptomatic intracranial hemorrhage
